# Pathogenic Infections during Pregnancy and the Consequences for Fetal Brain Development

**DOI:** 10.3390/pathogens11020193

**Published:** 2022-01-31

**Authors:** Sukanta Jash, Surendra Sharma

**Affiliations:** Department of Pediatrics, Women and Infants Hospital-Warren Alpert Medical School, Brown University, Providence, RI 02905, USA; sjash@wihri.org

**Keywords:** autism spectrum disorder, viral infections, uterine immune activation, cytokine storm, fetal brain development, sexual dimorphism, interleukin 17, regulatory T cells, T helper 17 cells

## Abstract

Pathogens comprised of viruses, bacteria, gut microbiome, and parasites are a leading cause of ever-emerging diseases in humans. Studying pathogens for their ability to cause diseases is a topic of critical discussion among scientists and pharmaceutical centers for effective drug development that diagnose, treat, and prevent infection-associated disorders. Pathogens impact health either directly by invading the host or by eliciting an acute inflammatory immune response. This paradigm of inflammatory immune responses is even more consequential in people who may be immunocompromised. In this regard, pregnancy offers an altered immunity scenario, which may allow the onset of severe diseases. Viruses, such as Influenza, HIV, and now SARS-CoV-2, associated with the COVID-19 pandemic, raise new concerns for maternal and fetal/neonatal health. Intrauterine bacterial and parasitic infections are also known to impact pregnancy outcomes and neonatal health. More importantly, viral and bacterial infections during pregnancy have been identified as a common contributor to fetal brain development defects. Infection-mediated inflammatory uterine immune milieu is thought to be the main trigger for causing poor fetal brain development, resulting in long-term cognitive impairments. The concept of in utero programming of childhood and adult disorders has revolutionized the field of neurodevelopment and its associated complications. Recent findings in mice and humans clearly support the idea that uterine immunity during pregnancy controls the health trajectory of the child and considerably influences the cognitive function and mental health. In this review, we focus on the in utero programming of autism spectrum disorders (ASD) and assess the effects of pathogens on the onset of ASD-like symptoms.

## 1. Introduction

Viral and bacterial infections during pregnancy have been associated with an array of adverse pregnancy outcomes, including spontaneous abortion, premature birth, stillbirth, intrauterine growth restriction, and fetal neurological defects [1,2,3,4]. During pregnancy, the mother and her semi-allogeneic fetus coexist, despite the robust maternal immune repertoire at the maternal–fetal interface [5]. Maternal and placental mechanisms must work in tandem to protect the fetus from pathogens and induced inflammation. This maternal–placental communication must also be required to establish an immune–brain axis [6]. The placenta is the first chimeric immunological organ to emerge, bridging the maternal and fetal circulations, controlling the in utero environment to harmonize the mother’s immune system throughout fetal development. Pregnancy also creates a window of infection risk. In the early stages of pregnancy, invading trophoblasts engage directly and intimately with the mother’s uterine immune cells. More importantly, the syncytiotrophoblast layer (STB) of the placental villi bathes in maternal blood, exposing it to circulating nutrients for fetal health and to pathogens for detrimental effects [7]. The maternal–placental interface also provides a synchronous interplay between the maternal vascular endothelium and resident immunocompetent cells. However, viruses have evolved intricate strategies to overcome placental control and activate uterine inflammatory immune responses to cause detrimental effects.

The mother’s immune responses to a pathogen and health rather than the ensuing disease itself may be responsible for the abnormalities in fetal brain development [8]. A pregnancy compatible immune environment controlled by nutrition, physiological stress, and hormonal regulation plays a key role in healthy brain development. Recent research links viruses and bacteria with neurodevelopmental anomalies, such as autism spectrum disorders (ASD) [9,10,11,12]. This suggests that numerous risk factors may be at play in determining whether or not a mother’s stress and immunological dysregulation during pregnancy are linked to the development of ASD. The behavioral, pathological, and epigenetic impacts of these interactions, as well as the possible mitigating factors, are just now beginning to be understood [13,14]. Understanding why male offspring is more likely to be diagnosed with ASD requires a better understanding of regulatory mechanisms at the placenta level. Since 61% of ASD cases remain an unexplained variety, a better understanding of these mechanisms can lead to a tailored therapeutic strategy, at least for the unexplained and male-centric development of ASD [15,16]. 

The prenatal innate and adaptive immunological milieu is thought to be implicated in ASD [17]. Some key in utero inflammatory indicators have been linked to ASD. According to the research in animal models, the pro-inflammatory phenotype of uterine, not systemic, immune activation is often associated with cognitive deficits and stereotypic behavior [18]. This raises a pertinent question of how uterine immune activation (UIA) during pregnancy can result in the onset of ASD in the offspring. Recent research suggests that the propagation of T helper 17 (Th17) cells and their effector cytokine IL-17A at the maternal–fetal interface are involved in the development of UIA-mediated ASD [19,20]. In a population of ASD children, elevated IL-17A blood levels have been observed to correspond with their phenotypic severity [21]. IL-17A levels have also been found to be increased in the UIA mouse models of ASD [19,20]. The prevention of ASD-associated behavior in the offspring by specific IL17A antibody treatment has also been observed [22]. IL-17A dysregulation may therefore have a central role in the development of ASD. Recent observations further suggest that UIA directly affects GABaergic interneurons, which may result in the reduction in cell numbers, redistribution across cortical regions and layers, changes in morphology and cellular activity, and altered transcriptomes in the cortex [23]. This raises a question about whether IL-17A is responsible for cortical architectural defects. Taken together, it is proposed that UIA-mediated effects in a developmental stage-dependent manner contribute to an immune origin of cognitive impairment in the offspring, particularly in male offspring. In this review, we focus on the timing of infections, the origin of Th17 propagation as no such cells are significantly present at the maternal–fetal interface during normal pregnancy, altered transcriptomes, and the cortical architecture impairment and its association with ASD-like social behavior.

## 2. Pathogenic Infection during Pregnancy and Its Relation to Neurodevelopmental Disorders

Increasing evidence has connected maternal bacterial infections to the abnormal neurodevelopmental process. Among offspring, the development of psychosis was substantially linked to maternal bacterial infections during pregnancy. Studies have shown that infections in the mother’s respiratory tract, such as sinusitis or tonsillitis, pneumonia or pyelonephritis, or bacterial venereal disease, are linked to a twofold increase in the child’s neurodevelopmental risk. Physical changes and immunological suppression during pregnancy result in an increased risk of developing urinary tract infections (UTIs), as well as bacterial vaginosis (BV) [24]. Infections during pregnancy and during fetal development, on the other hand, can be extremely dangerous, and untreated infections have been linked to serious neurodevelopmental abnormalities in children. An elevated risk of ASD was shown to be associated with bacterial infections, which accounted for the majority of illnesses discovered during a hospital stay [25]. Maternal bacterial infection during pregnancy may produce long-term alterations in the structure and function of the fetal brain, as demonstrated by animal studies. The exposed offspring showed behavioral, neurochemical, and neurophysiological abnormalities similar to psychotic disease [26].

To date, pathogens that have been considered in the context of fetal development include the classic group of teratogenic pathogens, referred to as “TORCH” *Toxoplasma gondii*, and others, such as Treponema palladium, Rubella virus, Cytomegalovirus (CMV), and herpes simplex virus (HSV) [27]. However, other viruses and bacteria make a broader group of pathogens, including Influenza virus (H1N1), Zika virus, *Plasmodium falciparum*, and now SARS-CoV-2, among the growing list of pathogens that may impact fetal brain development [28,29,30,31]. Some of the viruses, such as Rubella virus and CMV, are associated with congenital viral disease. CMV and ZIKV infections during pregnancy have been linked to microcephaly. RNA viruses, primarily of the Flaviviridae family are known to be neurotropic pathogens. The most recent outbreak was caused by Zika virus (ZKV), which causes microcephaly and ASD [32]. Other related flaviviruses, such as the West Nile virus (WNV), Japanese Encephalitis virus (JEV) and Dengue virus (DENV), have much lower rates of vertical transmission, but are neurotropic. Maternal West Nile virus (WNV) infection causes encephalitis, depression, memory loss, and motor dysfunction. Prenatal JEV infection causes encephalitis, paralysis, seizures, the inability to speak, memory loss, impaired cognition, and other mental disorders. Additionally, maternal DENV infection can cause encephalopathy, severe scattered encephalomyelitis, neuritis brachialis, stroke, neuro thalamic complications, myelitis, and acute hypokalemic disorders [33]. Viruses can breach fetal brain barriers in a variety of ways once they cross the placental barrier. It is possible for viruses, such as polio and mumps, to directly penetrate the brain parenchyma, resulting in symptoms, such as aseptic meningitis and paralysis [34,35]. A prominent cause of neurodevelopmental abnormalities in infants is the herpes simplex virus (HSV-1), one of the most common DNA viruses that affects pregnant women at various stages of pregnancy. HSV-2 infection, on the other hand, is far less prevalent but has been linked to ASD [36,37]. Neurosensory damage, localized encephalitis, microcephaly, paralysis of specific muscles, mental impedance, and autism spectrum disorder are all caused by Cytomegalovirus (CMV), another herpes viridae DNA virus [38]. Rubella virus infection during pregnancy causes congenital Rubella syndrome, microcephaly, panencephalitis, and autistic spectrum disease [39].

Several of these viruses may not cross the placental barrier. On the other hand, RNA containing viruses, such as Influenza virus and SARS-CoV-2, which are now a major public health burden but have a low propensity for vertical transmission, are considered to be potent effectors of fetal development anomalies, particularly brain development and diseases, such as ASD [28,30,31]. It is currently unclear how viral infections that do not significantly infect the placental cells or cross the placenta can impair the fetal development, particularly neural development. It has been shown that maternal viral infections do not have to cross the placental barrier in order to disrupt the fetal development [40,41]. In this regard, animal models have proven to be very useful in helping us better understand this complex scenario [19,22]. These models have revealed the possible maternal immunological pathways and cytokines that may be relevant for behavioral abnormalities in the offspring. Unscheduled propagation of Th17 cells at the maternal–fetal interface and availability of IL-17a have recently been found to be the primary cause of the fetal brain development and behavioral defects associated with viral infections mimicked by the double-stranded RNA analog polyinosinic–polycrytidylic acid poly(I:C) in pregnant mice [19,22]. Overall, these findings show that the immune dysregulation in pregnant women affects the fetal growth development and alters brain structure. The evidence from animal experiments suggests that normal fetal brain development depends on normal placental development and immune tolerance.

The hemochorial placenta is thought to be evolved by taking cues from the viral strategies to avoid host rejection. One of the crucial adaptations from viral infections is to integrate the genetic sequence of the enveloped protein ERVW-1. ERVW-1 is an endogenous retroviral element and encodes cell–cell fusion protein syncytium-1 during placental development to establish the STB layer. STBs lack gap junction and create defense barrier against invading pathogens [42]. It is noteworthy that the placenta uses this syncytial barrier to prevent the transmission of some viruses, but allows other viruses to reach the fetal circulation. The mechanism of pathogen transmission varies and depends on specific genetic requirement. The threshold of maternal viremia is critical for the hematogenous spread of viral pathogens within the placenta [3,43]. The importance of the viral threshold can be explained by the fact that many recurrent viral infections, such as HSV, only cause minimum placental infection and fetal damage. The stages of placentation also determine the susceptibility of viral infections [4,44]. The pathogenicity of adenovirus and herpes simplex viral infection is significantly reduced as trophoblasts differentiate into multinucleated STB, resulting in the sudden reduction in viral receptors. As the STB layer of the placenta forms a continuous barrier and contacts maternal blood, many viruses, such as HIV, CMV, and SARS-CoV-2, bind to the receptor present on the maternal side of the placenta and utilize antibody-dependent enhancement (ADE) to cross the STB layer [45,46,47]. 

## 3. Autism Spectrum Disorders

Early social impairment, repetitive behavior, communication difficulties, and learning and speech difficulties are all hallmarks of autism spectrum disorders (ASD). Social cognition and perception are poor in people with autism, executive dysfunction is very common, and information processing is impeded [48]. In the development of ASD, genetic factors play a significant influence, but the early exposure to environmental disruptors during brain development considerably contributes to its severity. It has been demonstrated in several studies, including systematic and meta-analyses, that infections during pregnancy increase the chance of ASD. There are many factors that contribute to ASD, including single gene mutations (SNPs), polymorphisms, and minor effect common variants [48,49]. According to the World Health Organization (WHO), ASD affects approximately 1 in every 160 children worldwide [50]. On the other hand, in the United States, the rate is astoundingly high with 1 in 54 children being affected.

The prevalence of ASD is 4–5 times higher in boys than in girls. In certain studies, there is a lower female to male ratio (1:3), indicating that females with ASD may be underdiagnosed compared to males [15,16]. It is interesting to note that the prevalence of ASD in siblings ranges from 6 to 24%. Compared to a younger female sibling (4%), a younger male sibling of an older male child with ASD is affected by more than 13%, while the ASD concordance rate increases dramatically in younger male siblings born after a female child with ASD (17%) [15]. The siblings of children with ASD or associated neurodevelopmental symptoms are more likely to have ASD or other associated neurodevelopmental symptoms than the siblings of children without ASD. However, even though they share the same DNA pool, identical twins with ASD frequently exhibit significant disparities in prevalence as well as severity of symptom manifestation. If one identical twin is diagnosed with ASD, the likelihood of the other identical twin being diagnosed with ASD increases by 60–90%. However, the rate of ASD concordance amongst fraternal twins is also quite significant [51]. The prevalence of ASD among same-sex fraternal twins is 34%, compared to 18% in opposite-sex fraternal twins [51,52].

## 4. Is the Gestational Timing of the Infection Key to the Development of ASD-like Brain Disorders?

**Figure 1:**
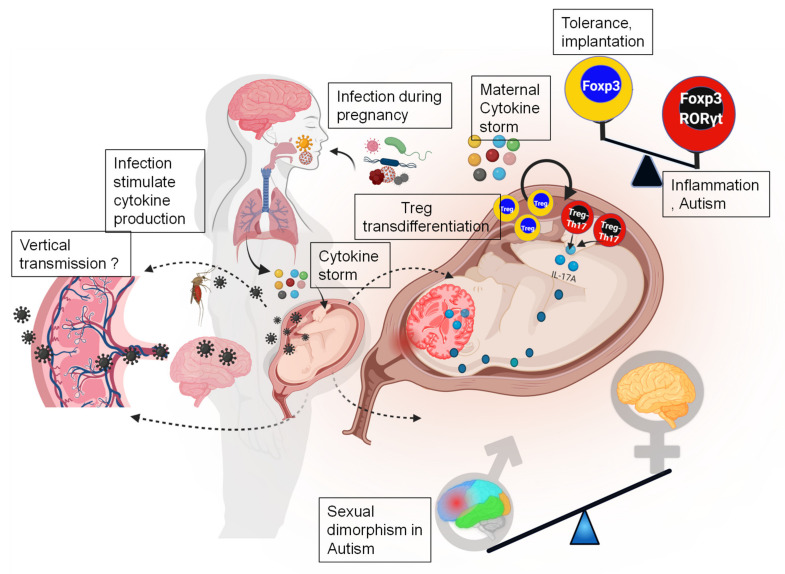
Uterine immunity-based multistep mechanism for autism: schematic representation of how viral infections affect the fetal neurodevelopment. Viral infections can affect the development of the fetal brain in both direct (ascending and vertical transmission), as well as immune-mediated cytokine storm syndrome. In some cases, lung epithelium infection, such as in the case SARS-CoV-2, results in cytokine storm syndrome and contribute to brain development defects. Another mechanism of immune activation occurs at the intrauterine level, where infections and other events can initiate a detrimental immune response. Immune activation at the maternal–fetal interface can also result in a localized cytokine storm. This cytokine storm disturbs the balance between uterine Tregs and Th17 towards a hybrid inflammatory Tregs–Th17 phenotype. These cells release IL-17, which crosses the placenta and increases the expression of the IL-17 receptor in the offspring’s brain. This results in sexually dimorphic cortical and behavioral defects in the progeny, with the male offspring being more affected than the female offspring. Additionally, TORCH (Toxoplasma gondii, other, Rubella virus, Cytomegalovirus, and herpes simplex virus) pathogens, including ZIKV, can access the intra-amniotic compartment through multiple mechanisms. Viruses that cause neurotropism and a localized cytokine storm in the developing fetus can affect brain development.

Pregnancy can be illustrated by three distinct stages: implantation, gestation, and parturition. The gestational period can be further divided into the temporal windows of placentation, neurogenesis, development of the functional blood–brain barrier, and birth. For example, in mice, placentation can be fully evaluated on the ninth day of pregnancy, followed by neurogenesis on the eleventh day of the pregnancy [53]. The pathogenesis of neurodevelopmental disorders, such as autism, is assumed to begin during early- to mid-fetal cortical development [54]. This human cortical developmental window (second trimester) overlaps with mouse corticogenesis at gestational days 11–14.5 [55,56] (Figure 1). Thus, understanding the delicate balance of neuronal subpopulations at this stage is critical for dissecting the events that cause brain developmental defects leading to syndromes, such as ASD. In humans, while no overall association between admissions of pregnant women for infection and development of ASD was reported, a subgroup analysis found a significant association between second trimester antenatal admission for bacterial infection and the subsequent development of ASD [57,58]. This gestational age timing can be easily proved by using animal models. We propose that there is a very strong biological reason for this gestational window for the onset of ASD-like cognitive syndromes.

Neocortical neurons are formed in the human cortex during early pregnancy through 18 weeks of gestation [53]. Post-mitotic neurons travel outward from the ventricular zone (VZ) to different cortical layers near the end of the proliferation stage, when they produce axons and dendrites and begin to make synaptic connections [59,60]. Neuronal progenitors divide rapidly in the mouse cortex between E11.5 and E14.5 and continue to divide at a lesser pace until delivery [61]. Six neocortical layers are formed as a result of these finely orchestrated patterns of growth [59,61]. In mice, the gestational age window, E11.5-E14.5, indicates a progenitor-driven phase, and at birth (P0), all six neocortical layers have grown. Neuronal stem cells in the VZ, intermediate progenitors in the subcellular ventricular zone (SVZ), and radial glia in the cerebral cortex divide symmetrically or asymmetrically around E11.5 to form more intermediate progenitors or pyramidal neurons [62,63]. Terminally developed neurons move radially from E12.5 to E14.5 to produce cortical layers and lamina from the inside out. Depending on the neuronal cell fate, these neurons are characterized by distinct transcription factors, such as CTIP2, SATB2, and TBR1. The transient stage-specific expression of these transcription factors regulates the distinct axonal projections, such as corticothalamic (CThPN), subcerebral (SCPN), and callosal projection neurons [52,61,62]. During this period, the Wnt–catenin and mTOR pathways regulate the size, distribution, and position of the cortical regions [64,65]. Except for the SVZ, hippocampus dentate gyrus, and cerebellar cortex, the brain enters a condition of replicative quiescence at the start of postnatal life [52,58,62]. Given the chronology and trajectory of human brain development, any infection, trauma, or treatment opportunity during the first 12–16 weeks of pregnancy may have a significant impact on cortical neurogenesis. In this regard, viral or bacterial infections during this window of pregnancy are likely to impact cortex architecture and transcriptional choreography, cognitive growth, and social behavior.

## 5. Cytokine Storm at the Feto–Maternal Interface during Uterine Immune Activation in Relation to ASD

A number of cytokines have been linked to uterine immune activation (UIA) and the severity of disease phenotypes [66] (Figure 1). Several factors influence the cytokine threshold, including the gestational age, pathogen type, and time since the onset of UIA. IL-6 and IL-17a, two cytokines, appear to play a critical role in the behavioral disorders of the offspring born to UIA-affected mothers [20,67]. As Cre-mediated deletion of the IL-6 receptor (IL-6R) in the placental trophoblast entirely abrogates fetal brain injury and hence behavioral deficits in the offspring, the trophoblast appears to be the key source and target of IL-6 [68]. A single-dose maternal injection of recombinant IL-6 without additional pathogen mimics can successfully recapitulate ASD- and schizophrenia-related behavior [69]. In addition, the co-administration of poly I:C with monoclonal antibodies neutralizing IL-6 or IL-17a fail to induce ASD-like behavioral abnormalities [20,69]. No behavioral impairments were observed in the offspring of the IL-6 knockout mice that had been exposed to an immunological challenge [69]. As a result, it appears that even a brief activation of the IL-6 pathway in the placenta during the second trimester can be harmful. Recent research suggests that induction of IL-17a occurs downstream of IL-6 induction [19,22] (Figure 1). Recent observations on UIA-mediated effects suggest that the microbiome of the UIA-exposed mother’s gut exerted significant influence on the brain development and behavior of their offspring [67,70]. On the other hand, high levels of TNF-α were not directly related to the onset of ASD-like features as its levels remained high post-UIA induction [67,71]. The behavioral abnormalities caused by UIA in the offspring could not be reversed by TNF-α blocking during UIA. Recombinant IL-2, similar to IL-6 and IL-17a, can generate many ASD-like neurodevelopmental behavioral impairments when injected during mid-pregnancy. The anti-inflammatory cytokine, IL-10, is likewise elevated in the maternal serum and placenta after UIA, suggesting the existence of an internal immune system counterbalance. The effects of IL-6 and IL-17a may be mitigated to some extent by maternal IL-10 [72]. While the overexpression of IL-10 in the absence of an inflammatory insult was linked with deficits in spatial and associative learning, transgenic overexpression of IL-10 in the late pregnancy UIA model reduced the development of schizophrenia-like behavioral abnormalities [73,74,75].

## 6. Sex Differences in Response to UIA

Males and females have different brain transcriptomes, especially during development [76]. During prenatal development, more genes displayed sex-biased expression than during postnatal life in a transcriptome investigation of the human brain. Genes on the Y chromosome were responsible for the most significant changes. According to these profiles, the fundamental variations in brain development between males and females occur during the perinatal period and are mostly influenced by the differential expression of genes on sex chromosomes, although many additional genes on autosomes may also contribute [77,78]. There are two-to-four times as many boys diagnosed with ASD as girls, although the reasons for this disparity remain unknown. However, despite the fact that this male preponderance is not specific to ASD, it has been viewed as a crucial indication to identifying the underlying cause. The male preponderance does not appear to be directly linked to genetic factors, as the sex-skewed expression of neurodevelopmental risk genes has not been identified [79,80]. It is also possible that the diagnosis may be affected by the fact that males with ASD tend to demonstrate more expressive behavior (for example, repetitive and aggressive behavior), whilst females tend to display more affectionate behavior (for example, emotional symptoms). Males are more susceptible to genetic mutations and environmental stressors than females [81]. Various inflammatory stressors to the fetus in the womb have been shown to affect the susceptibility of male and female fetuses differently [81] (Figure 1). These discrepancies might be explained by gender variations in the placental responses to pathogen-induced UIA, fetal brain anatomy and function, and immune cell characteristics [82,83,84,85,86]. Additional findings suggest that the male brain is comparatively significantly influenced by the innate immune system activation [85]. Neuron–glial interactions in the brains of patients with ASD may be sexually dimorphic [87]. Thus, it will be important to conduct longitudinal brain transcriptome investigations to determine if sexually dimorphic pathways in microglia have a role in the development of ASD.

## 7. A Fine Balance between Tregs–Th17 Cells and IL-17A Pathway in ASD

A growing body of evidence suggests that Th17 cells and their cytokine IL-17A play a role in ASD [19,20,22]. There were enriched copy number variations in the gene encoding IL-17A associated with ASD in a genome-wide analysis [88,89]. IL-17A has been shown to be increased in the blood of certain persons with ASD and has been linked to the severity of their behavioral symptoms. The placenta controls the mother’s immune challenges to the fetus, allowing the child to develop a healthy immune system. There is evidence that the uterine immune cells are triggered to generate IL-17A in response to poly(I:C)-induced UIA [19,20] (Figure 1). The mRNA levels of IL-17A are elevated in the placenta. Poly(I:C)-treated pregnant mice show increased population of Th17 cells and elevated IL-17A levels, although this response was unique to cells separated from the placenta. It is unclear how and when Th17 cells are generated, because there are few or no Th17 cells at the maternal–fetal contact in a normal pregnancy. Our unreported findings imply that uterine immune activation during a key period of pregnancy may cause regulatory T cells (Tregs) to trans-differentiate into Th17 cells, affecting fetal brain development and inducing an ASD-like behavioral phenotype in the offspring (Figure 1). During pregnancy, the placenta’s regulatory and pro-inflammatory T-cell activity must be carefully coordinated [90,91]. UIA alters the cytokine milieu, increasing IL-6 levels, to favor the activation of maternal Th17 cells. Furthermore, UIA at E12.5 enhanced the abundance of the uterine hybrid CD4^+^CD25^+^ Foxp3^+^RORγT^+^Tregs (Tregs-Th17), indicating a pathological transition of Tregs toward the Tregs–Th17 phenotype and the generation of hybrid Tregs capable of secreting IL17a. Functional plasticity exists between Th17 and Foxp3^+^ Tregs cells [92,93]. In autoimmune arthritis, the transdifferentiation between these two cell types was observed [94]. Maternal Th17 cells release effectors, such as IL-17A, which may influence fetal development indirectly by regulating placental function and producing intermediary soluble factors that enter fetal circulation, or directly by transport into the fetus. Maternal Th17 cells have been shown to be transferred to the fetus [95,96,97].

## 8. Conclusions and Future Perspective

In summary, pathogenic infections and brain disorders, such as ASD, appear to be immunologically connected, particularly activation of uterine immunity. Although ASD and other cognitive and social behavior diseases are extremely complicated neurodevelopmental disorders with no definite etiology, gestational stress and pathogenic infections are emerging as causative factors. It is noteworthy to mention that these triggers may impact varying outcomes depending on their gestational window of exposure. While genetics play a significant role in the programming of ASD and other brain disorders, not all ASD and brain disorders cases can be explained by genetic factors. Evidence is accumulating that environmental variables, including pathogens, are key contributory triggers. Future research is also needed to better understand the link between the non-genetic triggers and the gender of the offspring, as evidence shows that the male offspring are more susceptible to the disease. One could argue for the involvement of systemic immunological/inflammatory events; however, changes in the immunological and epigenetic systems found in the peripheral system are not necessarily indicative of changes in the brain. It is widely expected that a single-hit model does not represent the whole range of brain and behavioral alterations associated with ASD and other brain disorders. As proposed in this review, pathogenic infections during pregnancy, particularly during a temporal window, may converge with other underlying conditions to induce fetal brain developmental defects, resulting in ASD-like syndromes. In future investigations, it is critical to understand the molecular signaling pathways that are activated by pathogenic infections and IL-17a activation in ASD.

## Abbreviations

IL17a	Interleukin 17a isoform
IL-6	Interleukin 6
Th17	T helper 17 cells
ASD	Autism spectrum disorders
UIA	Uterine immune activation
LPS	Lipopolysaccharide
Poly I:C	Polyinosinic polycytidylic acid
SARS-CoV-2	Severe acute respiratory syndrome coronavirus 2
COVID-19	Coronavirus disease 2019
ZKV	Zika virus
CMV	Cytomegalovirus
ERVW-1	Endogenous retrovirus group W member 1 (Syncytin-1)
Tregs	Regulatory T cells
TNFα	Tumor necrosis factor alpha
STB	Syncytiotrophoblast
